# Metagenomic characterization of the microbiomes in five different body habitats of otherwise healthy individuals with periodontal disease

**DOI:** 10.3389/fcimb.2023.1257816

**Published:** 2023-09-13

**Authors:** Sujin Oh, Hyo-Jung Lee, Kyoung Un Park

**Affiliations:** ^1^ Department of Laboratory Medicine, Seoul National University College of Medicine, Seoul, Republic of Korea; ^2^ Department of Periodontology, Section of Dentistry, Seoul National University Bundang Hospital, Seongnam, Republic of Korea; ^3^ Department of Laboratory Medicine, Seoul National University Bundang Hospital, Seongnam, Republic of Korea

**Keywords:** oral microbiome, blood microbiome, stool microbiome, periodontal disease, otherwise healthy

## Abstract

**Introduction:**

Recent studies have proposed several plausible mechanisms supporting the association between periodontal disease and systemic disease. However, characterizing the microbial communities in individuals with periodontal disease before onset of other diseases is an important first step in determining how the altered microbial state contributes to disease progression. This study established microbiome profiles for five body habitats of carefully selected, otherwise healthy individuals with periodontal disease.

**Methods:**

Blood, oral (buccal mucosa, dental plaque, and saliva), and stool samples were collected from ten healthy subjects with periodontal disease. Using 16S rRNA metagenomics, the taxonomic and functional compositions of microbiomes were investigated.

**Results:**

The most predominant phylum in blood and stool was Bacillota. Pseudomonadota accounted for the largest proportion of microbes in the buccal mucosa and saliva, whereas Bacteroidota were the most prevalent in dental plaque. Differential abundance analysis revealed that 12 phyla and 139 genera were differentially abundant between body habitats. Comparison of alpha diversity showed that the blood microbiome has the most diverse community close to neither oral nor stool microbiomes. We also predicted the functional configurations of the microbiome in otherwise healthy subjects with periodontal disease. Principal coordinate analysis based on functional abundance revealed distinct clustering of the microbial communities between different body habitats, as also observed for taxonomic abundance. In addition, 13 functional pathways, including lipopolysaccharide biosynthesis, glutathione metabolism, and proteasome, showed differential expression between habitats.

**Discussion:**

Our results offer insight into the effects of the microbiome on systemic health and disease in people with periodontal disease.

## 1 Introduction

Understanding the interactions between the host and microbial communities in varied environments in the human body is crucial to achieving insights into their roles in human health and disease. With the advent of high-throughput next-generation sequencing, many studies have sought disease-associated microbiomes using metagenomics analyses, involving the sequencing and analysis of genes from whole communities rather than from individual genomes (Chiu and Miller, 2019). The best studied of these is the association between the gut microbiome and the pathogenesis of gastrointestinal disorders, such as inflammatory bowel disease (Manichanh et al., 2012) and several types of cancer (Meng et al., 2018), and many cardiometabolic implications, such as hypertension (Avery et al., 2021) and type 2 diabetes (Sharma and Tripathi, 2019).

The oral microbiome also has practical implications regarding disease, as it plays important roles not only in the oral cavity but also in the whole body. The oral cavity contains multiple interacting microhabitats, including the buccal mucosa, subgingival plaque, and saliva, which lead to unique microbial compositions that are different from other body habitats (Welch et al., 2020). Moreover, microbial dysbiosis in the oral cavity has been implicated in the development of many non-communicable diseases. In addition to infective endocarditis and aspiration pneumonia, there is evidence that periodontal disease is closely linked to a variety of non-communicable diseases, including cardiovascular disease (Sanz et al., 2020), Alzheimer’s disease (Dioguardi et al., 2020), and certain types of cancer (Fitzpatrick and Katz, 2010; Dizdar et al., 2017).

To better understand the association between periodontal disease and health, it is necessary to obtain insights into the microbial communities in individuals with periodontal disease in the absence of other diseases. Previous efforts have mainly focused on the microbiomes in the niches of the oral cavity, and there have been fewer studies on the gut microbiome. In one study, individuals with chronic periodontitis showed lower alpha diversity in the stool microbiome but the difference was not significant (Lourenςo et al., 2018). To the best of our knowledge, there have been no previous studies of the blood microbiome in subjects with periodontal disease.

Blood in the closed circulation system has been considered a sterile environment, and it is a common belief that microbes are present in the blood only in case of sepsis. However, the concept of ‘sterile’ blood has been recently brought into question. The presence of a blood microbiome has been described in the setting of various non-communicable diseases such as cardiovascular events (Amar et al., 2013; Velmurugan et al., 2020), liver cirrhosis (Kajihara et al., 2019), or diabetes (Qiu et al., 2019; Velmurugan et al., 2020) without any clinical evidence of infection. In addition, the presence of microorganisms was confirmed in the blood of healthy donors.

Characterizing the baseline state of the microbiome is an important first step in determining how the altered microbial state contributes to disease progression. The Human Microbiome Project (HMP) funded by the National Institutes of Health has isolated and sequenced more than 1300 reference strains from 18 parts of the body in 300 healthy adults (Human Microbiome Project C, 2012a; Human Microbiome Project C, 2012b). However, that project, like most human microbiome studies, represents Western populations, although recent studies have elucidated geographic and ethnicity-specific variation (Gupta et al., 2017). Thus, more work needs to be done to characterize the disease-free state of the microbial communities of eastern populations.

In this study, we established microbiome profiles in various body habitats of otherwise healthy individuals of an East Asian population with periodontal disease. We examined the taxonomic and functional compositions of microbiomes in five different body habitats. An improved understanding of the microbiome communities in various body habitats will facilitate early detection of changes in the microbiome associated with disease states, preferably in preclinical stages, in otherwise healthy individuals with periodontal disease.

## 2 Results

### 2.1 Microbial diversity in five habitats of healthy individuals with periodontal disease

**Figure 1:**
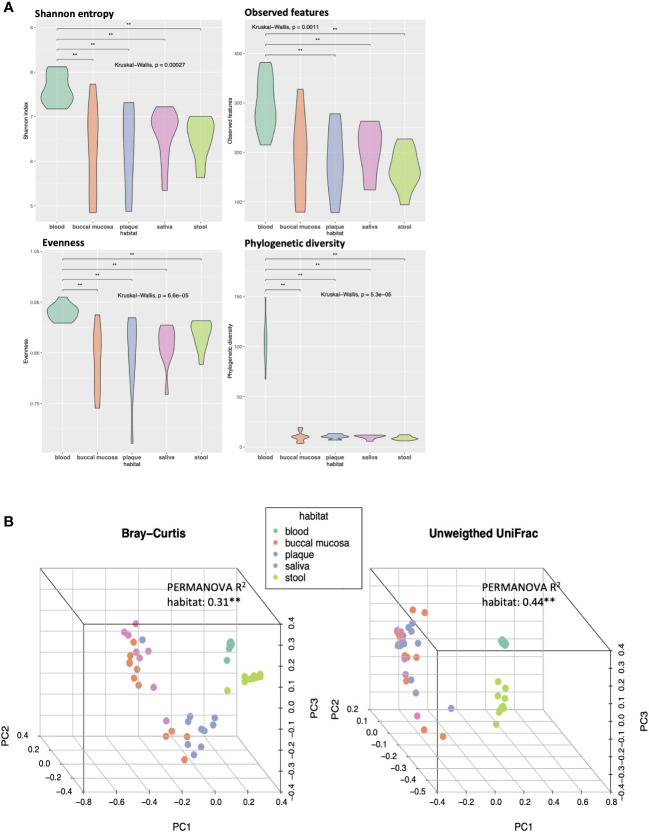
Differences in alpha and beta diversity of the microbiomes in five different habitats of otherwise healthy individuals with periodontal disease. **(A)** Violin plots representing each habitat’s microbial alpha diversity (Shannon entropy, observed features, evenness, and phylogenetic diversity). **(B)** Three-dimensional principal coordinate analysis (PCoA) plots of beta diversity indices (Bray-Curtis and unweighted UniFrac distance) showing the dissimilarities in microbial composition between different body habitats (**P < 0.01, PERMANOVA).

Alpha and beta diversity analyses were conducted to evaluate the differences in the diversity of microbial communities in blood, buccal mucosa, plaque, saliva, and stool samples (**Figure 1**). The blood microbiome showed the highest values of both richness and evenness in all indexes compared to other habitats (**Figure 1A**). To assess the dissimilarities of microbial composition between the five habitats, principal coordinates analysis (PCoA) was performed on the beta diversity matrices, PCoA of Bray-Curtis and unweighted UniFrac distance clustered the samples into blood, oral, and stool with distinct separation (**Figure 1B**; R^2^ = 0.31, *P* < 0.01 and R^2^ = 0.44, *P* < 0.01, respectively). In addition, significant differences in beta diversity were observed between the three habitats in the oral cavity (buccal mucosa, plaque, and saliva) using pairwise tests (*P* < 0.05, respectively; data not shown).

### 2.2 Taxonomic profiles of blood, oral, and stool microbiomes of healthy individuals with periodontal disease

**Figure 2:**
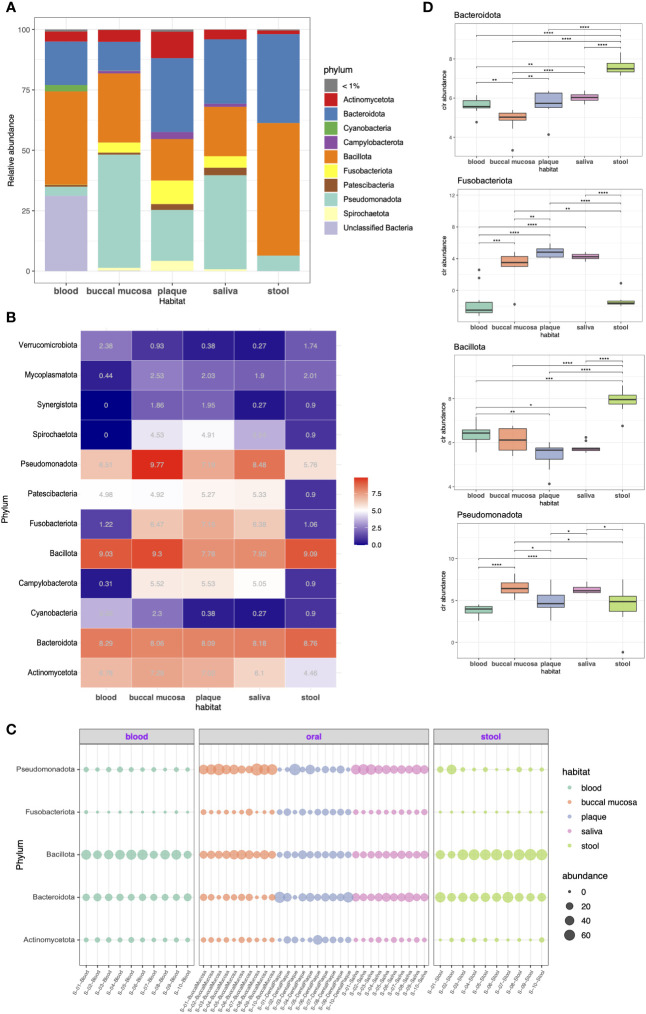
Taxonomic profiling of resident microorganisms in five different habitats of otherwise healthy individuals with periodontal disease at the phylum level. **(A)** Bar plot presenting the phylum-level composition of each habitat. Phyla representing less than 1% of the microbial community are indicated as <1%. **(B–D)** indicate the differentially abundant phyla between different body habitats confirmed by ANCOM-BC (FDR < 0.05). **(B)** Heatmap showing the bias-corrected abundance of differentially abundant phyla. **(C)** Bubble plot showing the relative abundance of five selected phyla in each sample. **(D)** Box plot of the centered log ratio (CLR)-transformed abundance of five selected phyla. The pairwise Wilcoxon’s signed-rank test was used to determine the significance of differences between each habitat (*P < 0.05; **P < 0.01; ***P < 0.001; ****P < 0.0001).

We investigated the taxonomic compositions of each microbiome at the phylum and genus levels. As shown in **Figure 2A**, the predominant phyla across the five habitats were Bacillota (formerly Firmicutes), Pseudomonadota (Proteobacteria), Bacteroidota (Bacteroidetes), and Actinomycetota (Actinobacteria), together constituting 64.3%, 92.2%, 79.7%, 89.9%, and 99.4% of the microbiome in the blood, buccal mucosa, dental plaque, saliva, and stool, respectively. Bacillota was the most abundant phylum in stool (54.8%), where it had higher abundance than it had in other habitats. Bacteroidota also had a high abundance in stool (36.7%); it was also the most dominant taxon in dental plaque (30.5%). Pseudomonadota, a major phylum of Gram-negative bacteria that includes many important human pathogens, was most abundant in the buccal mucosa (46.8%) followed by the saliva (38.9%). Several phyla, including Acidobacteriota (Acidobacteria), Gemmatimonadota (Gemmatimonadetes), Planctomycetota (Planctomycetes), and Rokubacteria, were only present in blood although each only accounted for less than 1% of the whole community. Overall, the blood microbiome showed the most differentiated phylum composition, and somewhat similar patterns were observed in the three habitats of the oral cavity.

Differential abundance (DA) tests were conducted using analysis of composition of microbiomes with bias correction (ANCOM-BC) to identify phyla and genera with significant differential abundance between habitats. Twelve phyla were determined to be differentially abundant phyla: Actinomycetota, Bacteroidota, Cyanobacteria, Campylobacterota (Epsilonbacteraeota), Bacillota, Fusobacteriota (Fusobacteria), Patescibacteria, Pseudomonadota, Spirochaetota (Spirochaetes), Synergistota (Synergistetes), Mycoplasmatota (Tenericutes), and Verrucomicrobiota (Verrucomicrobia). Particularly, Campylobacteroa and Spirochaetota were highly abundant in the oral cavity (most abundant in buccal mucosa) but were significantly less abundant in blood. The abundance of Patescibacteria was significantly lower in the stool microbiome, whereas the rest showed similar levels (**Figures 2B, C**). Moreover, we also performed pairwise comparison of the centered log-ratio (CLR)-transformed abundance using Wilcoxon’s test. The abundance of Bacteroidota differed between all groups except plaque and saliva. The abundance of Fusobacteriota also showed no difference between plaque and saliva, whereas it was more prevalent in the oral cavity than in blood or stool (**Figure 2D**).

**Figure 3:**
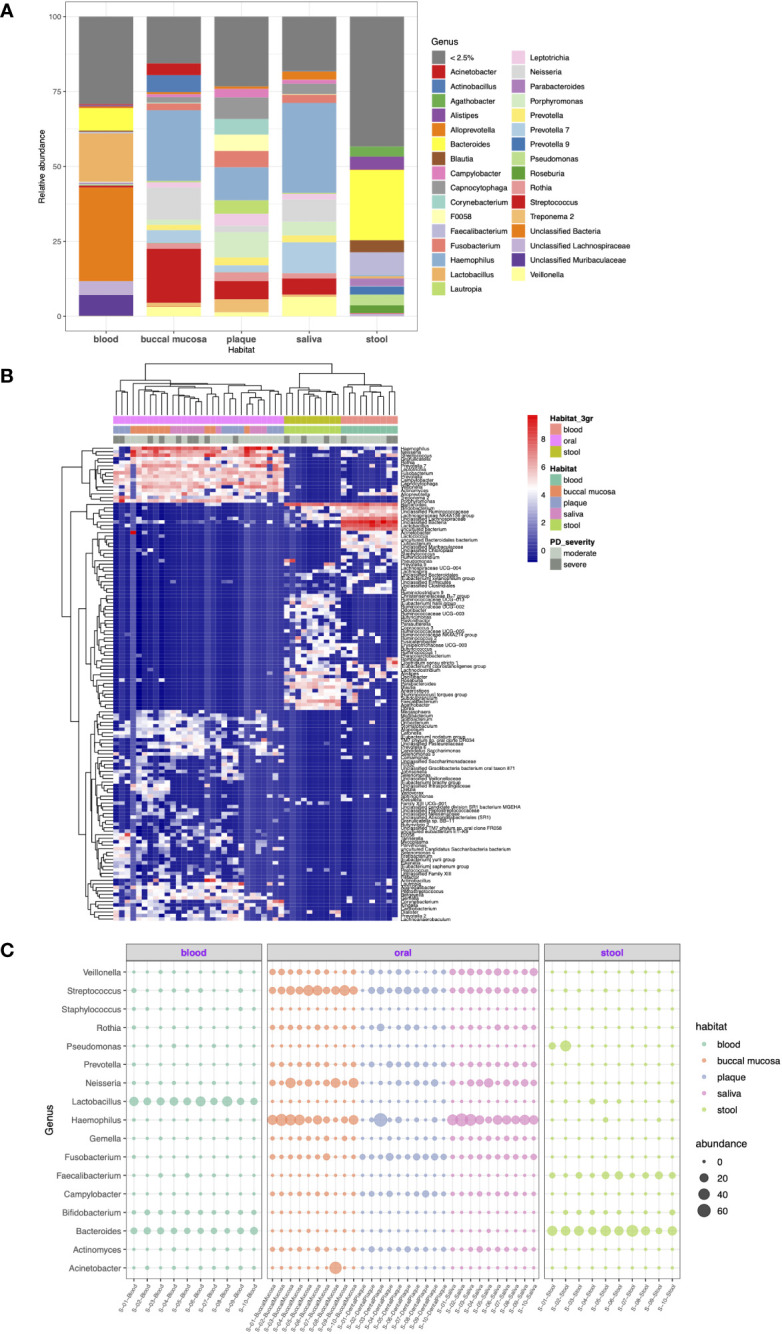
Taxonomic profiling of resident microorganisms in five different habitats of otherwise healthy individuals with periodontal disease at the genus level. **(A)** Bar plot showing the composition of genera in each habitat. Genera representing less than 2.5% of the microbial community are indicated as <2.5%. **(B, C)** show the results of ANCOM-BC emphasizing the differentially abundant genera between different body habitats (FDR < 0.05). **(B)** Heatmap generated by hierarchical clustering of the differentially abundant genera and apparent clustering between blood, oral, and stool microbiomes. **(C)** Bubble plot representing the relative abundance of 17 selected genera in each sample.

As shown in **Figure 3A**, 28.0%, 69.1%, 67.5%, 48.8%, and 48.9% of the total number of reads were represented by each of the seven most abundant genera in the blood, buccal mucosa, dental plaque, saliva, and stool microbiomes, respectively. Stool was characterized by a high relative abundance of *Bacteroides* (28%), while none of the samples from the oral cavity had *Bacteroides* among the seven most abundant genera. In blood, *Bacteroides* (7.4%) was the second most abundant genus, when the unclassified taxa were excluded. *Porphyromonas*, the genus of the major periodontal pathogen *Porphyromonas gingivalis*, had a relative abundance of 4.6% and 8.5% in saliva and dental plaque, respectively, but less than 1% in blood and stool.

With respect to DA at the genus level, 139 genera were determined to be differentially abundant between body habitats (FDR < 0.05). The hierarchical clustering of differentially abundant genera resulted in distinct clustering between blood, oral, and saliva microbiomes beyond other factors (**Figure 3B**). Particularly, *Fusobacterium*, *Prevotella*, *Rothia*, *Campylobacter*, and *Gemella* were highly abundant in the oral cavity but was significantly decreased in blood and stool. On the other hand, *Bacteroides* and *Faecalibacterium* were enriched in the stool microbiome compared to other habitats (**Figure 3C**).

### 2.3 Functional configuration of blood, oral, and stool microbiomes of healthy individuals with periodontal disease

**Figure 4:**
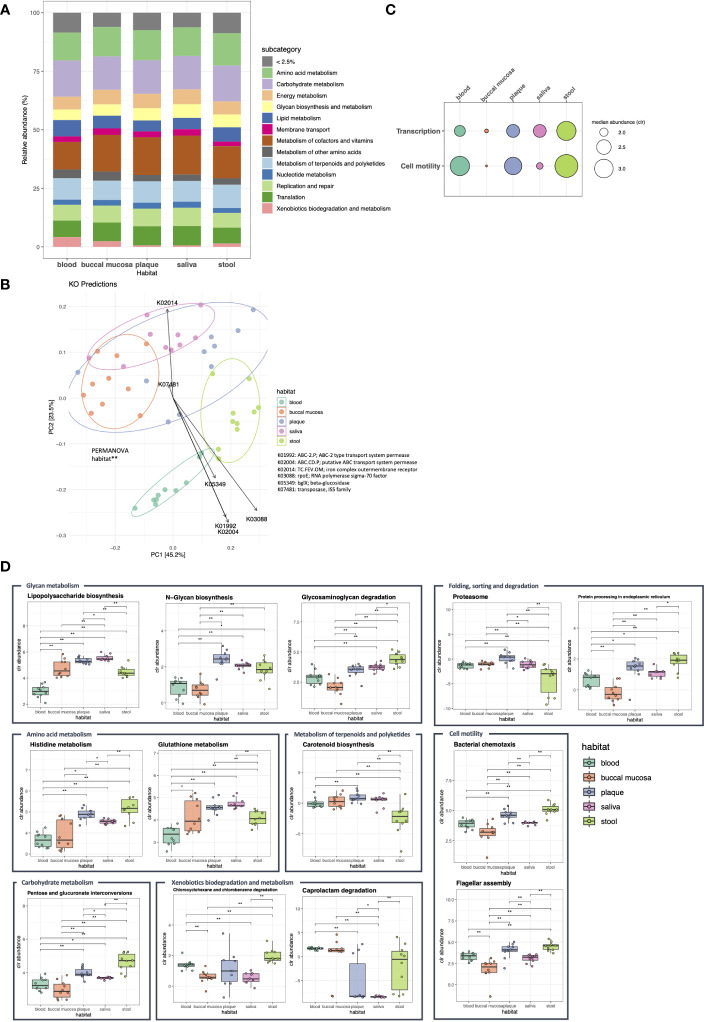
Functional compositions of the microbiome in five body habitats of otherwise healthy individuals with periodontal disease. Using the PICRUSt2 algorithms, the expression of metagenome functions was predicted based on the Kyoto Encyclopedia of Genes and Genomes (KEGG) orthologs (KOs), and the abundance of KEGG pathways was calculated at levels 1 and 2. **(A)** Bar plot showing the relative abundance of KEGG level 1 pathways. The pathways with an abundance of less than 2.5% are in dicated as <2.5%. **(B)** The biplot of the first two principal coordinate axes was generated using the Bray-Curtis distance computed from the KO profiles of each sample. The loading plot (arrows) indicates the KOs with the strongest contributions on the corresponding coordinates (**P < 0.05, PERMANOVA). **(C, D)** Show the pathways with differential expression between body habitats confirmed by ALDEx2 analysis (c: level 1; d: level 2). The box plots illustrate the centered log ratio (CLR)-transformed abundance of level 2 pathways in each habitat (*P < 0.05; **P < 0.01, Wilcoxon’s signed-rank test).

To elucidate the functional characteristics of the microbiomes in each habitat, Kyoto Encyclopedia of Genes and Genomes (KEGG) orthologs (KOs) were predicted and collapsed into pathways. When the functional abundance of each sample was estimated using the KEGG level 1 (upper level) pathway, the majority of metabolic pathways, including amino acid, carbohydrate, and lipid metabolism, were evenly distributed across the five habitats (**Figure 4A**; median: 12.5%, 14.3%, and 5.4%, respectively). Based on the KOs detected across the samples, we generated a PCoA biplot to visualize the vector overlay of the ortholog variables with the first two principal coordinate axes. PCoA of KO predictions revealed clear clustering of the microbial communities between different body habitats similar to the 16S rRNA marker genes (*P* < 0.01). Moreover, the top KOs with the highest loadings (contributions) on the corresponding coordinates included ABC transporter, rpoE, and beta-glucosidase (**Figure 4B**).

The differentially expressed pathways were determined using the generalized linear model with FDR < 0.05 and effect size > 1. Although most of the level 1 pathways showed no differences in abundance between body habitats, functions related to transcription were highly enriched in the stool microbiome, while those associated with cell motility were decreased in buccal mucosa and saliva (**Figure 4C**). Among the level 2 (lower level) pathways, 13 pathways showed differential expression among microbial communities in different habitats. N-glycan biosynthesis was enriched in dental plaque, while glutathione metabolism and lipopolysaccharide biosynthesis were enriched in the saliva microbiome. In addition, functional features, such as histidine metabolism and glycosaminoglycan degradation, were enriched in stool (**Figure 4D**).

## 3 Discussion

To the best of our knowledge, this is the first study to comprehensively evaluate the blood, oral, and stool microbiome in an East Asian population in relation to periodontal disease. We present the taxonomic and functional compositions of the five-habitat microbiome in otherwise healthy subjects with periodontal disease, providing insight into the state of microbiome in individuals with periodontal disease before the onset of any systemic disease.

Disturbances in the function and composition of the oral microbiome contribute to the pathogenesis of periodontal disease. The accumulation of biofilm and their interactions with immune response mediators cause inflammatory changes in the periodontal environment, driving the selection of anaerobic, acid-tolerating, and protein-dependent microbes and leading to chronic destructive inflammation (Kilian et al., 2016). Analysis of the functional composition of the microbiome revealed that lipopolysaccharide biosynthesis, glutathione metabolism, and protein degradation pathways were enriched in the oral cavity of individuals with periodontal disease. Recent studies have proposed several plausible mechanisms to support the systemic consequences of periodontitis. One of these is based on the entry of bacteria and immunological mediators from periodontal tissues into the circulation allowing them to act on other sites in the body (Reyes et al., 2013; Winning and Linden, 2017; Carrizales-Sepúlveda et al., 2018). Major periodontal pathogens, including *Porphyromonas gingivalis*, *Aggregatibacter actinomycetemcomitans*, and *Treponema denticola*, have been studied for their invasion through endothelial cells and resulting systemic inflammation (Foschi et al., 2006; Singhrao et al., 2015; Gholizadeh et al., 2017).

In addition, a few studies have demonstrated that periodontitis can induce dysbiosis of the gut microbiome in the long term, eventually establishing a disturbed gut microbiome commonly seen in individuals affected by systemic inflammatory diseases. Nakajima et al. reported that oral administration of *P*. *gingivalis* in mice significantly altered the gut microbiome, particularly with an increased proportion of phylum Bacteroidota and a decreased proportion of phylum Bacillota and increased levels of systemic endotoxins (Nakajima et al., 2015). Another 16S metagenomics study that compared the taxonomic profiles of the gut microbiome in cases of periodontal health and disease identified no significant differences in microbial composition groups, but the Bacillota/Bacteroidota ratio tended to increase in patients with periodontal disease (Lourenςo et al., 2018).

The dominant phyla or genera in each habitat were generally consistent with previous studies on healthy subjects, and differences in relative abundances between studies and different individuals may reflect physiological variation in the microbial communities among individuals (Dewhirst et al., 2010; Human Microbiome Project C, 2012b; Segata et al., 2012; Mark Welch et al., 2016; Gosiewski et al., 2017; Whittle et al., 2019). The stool microbiome mainly consisted of two predominant phyla, Bacillota and Bacteroidota, with Bacillota showing a higher abundance than Bacteroidota. The most predominant genus was *Bacteroides*, while significant enrichment of other genera, such as *Ruminococcaceae UCG-013*, *-002*, and *-003*, *Dorea*, *Agathobacter*, *Faecalibacterium*, *Odoribacter*, and *Parabacteroides*, was observed compared to other habitats.

The concept of the blood microbiome, especially in healthy individuals, remains inconclusive. The controversies revolve around two issues: microbial viability cannot be determined through sequencing-based profiling and microbial analysis of low-biomass samples is vulnerable to exogenous contamination (Potgieter et al., 2015; Castillo et al., 2019). Moreover, despite accumulating evidence for the existence of blood microbiome, the taxonomic composition of this microbiome remains to be elucidated. This study analyzed the microbial communities in the blood of systemically healthy subjects with periodontal disease. In contrast to previous studies of healthy donors in which Pseudomonadota or Actinomycetota was the most prevalent phylum, the most abundant phylum in the blood microbiome in our study was Bacillota followed by Bacteroidetota, Actinomycetota, and Pseudomonadota (Gosiewski et al., 2017; Qiu et al., 2019; Whittle et al., 2019). The blood microbiome was characterized by significant enrichment of the genera *Bifidobacterium*, *Lachnospiraceae NK4A136 group*, *Lactobacillus*, *Acinetobacter*, *Lactococcus*, *Cutibacterium*, *Staphylococcus*, and *Ruminiclostridium* (FDR < 0.05).

Furthermore, blood had the most diverse microbial community in terms of both richness and evenness. To date, the most plausible hypothesis for the origin of microbes in the blood is the translocation of microorganisms from other sources, mainly gut and oral cavity, during the human life cycle. In this regard, it is reasonable to assume that blood may contain adaptive microecosystems sensitive to environmental influences and exposure to novel microbial taxa, and therefore the blood microbiome would be highly dynamic (Castillo et al., 2019). Disruption of mucosal integrity in pathological states of the oral cavity could exacerbate microbial translocation, leading to more abundant and persistent microbes in the bloodstream. However, a clear explanation of this high diversity, with many unclassifiable taxa in blood, is still lacking.

A number of studies have supported the concept that the coevolution of microbes within the mouth has led to highly specific taxon–taxon interactions, limiting most microbes to the habitat type that is occupied by these neighbors (Dewhirst et al., 2010; Mark Welch et al., 2016). In contrast to blood and stool, the phylum Pseudomonadota was most prevalent in the buccal mucosa and saliva, whereas Bacteroidota was most prominent in dental plaque. At the genus level, the oral cavity exhibited significant richness of *Haemophilus*, *Neisseria*, *Streptococcus*, *Prevotella*, *Fusobacterium*, *Granulcatella*, *Rothia*, *Gemella*, *Leptotrichia*, *Campylobacter*, *Capnocytophaga*, *Veillonella*, *Actinomyces*, *Alloprevotella*, *Treponema*, and *Porphyromonas*. Particularly, the genera *Haemophilus*, *Prevotella*, *Fusobacterium*, and *Rothia* showed no site-specific differences within the mouth. The buccal mucosa was characterized by the highest abundance of *Streptococcus*, whereas the saliva was characterized by the highest abundance of *Veillonella*, and the abundances of *Neisseria* and *Gemella* were significantly decreased in dental plaque (**Supplementary Figure 1**).

According to PCoA based on KOs as well as ASVs in each sample, five habitats of the otherwise healthy subjects with periodontal disease showed distinct clustering. That is, each habitat, even those that were closely juxtaposed, had a distinct configuration of microbial communities with differences in abundance of taxa and functional pathways.

Our results outline the microbial profiles of saliva, buccal mucosa, dental plaque, stool, and blood in otherwise healthy individuals with periodontal disease. With increasing evidence of associations between periodontal diseases and various systemic diseases, our findings may provide insight into the effects of microbiomes on systemic health and disease in people with periodontal disease. Given the remaining controversies on the blood microbiome, further studies are warranted to determine the potential sources of these microbial genetic materials and their biological and clinical significance.

## 4 Materials and methods

### 4.1 Study design

Ten subjects with good general health aged between 28 and 58 years who visited the dental clinic of Seoul National University Bundang Hospital for periodontal treatment were recruited. Electronic medical records of the subjects, including routine laboratory blood test results and dental charts, were reviewed to investigate their history of diseases, including hypertension, diabetes, chronic infection, liver, kidney, cardiovascular disease, autoimmune disease, and malignant tumors, and history of antibiotic use within 6 months. The subjects did not manifest any symptoms or signs of infection at the time of sample collection with the exception of localized periodontal inflammation. The clinical features of patients are listed in **Supplementary Table 1**. Blood, buccal mucosa, subgingival plaque, saliva, and stool samples were collected from all subjects. The purpose of the study and the types of samples required were described to all potential participants. Following a questionnaire survey for pre-screening of potential study participants, and further assessment of eligibility, the subjects who provided written informed consent were included in the study.

This study was approved by the Institutional Review Board (IRB) of Seoul National University Bundang Hospital and was conducted in accordance with the Declaration of Helsinki (IRB number: B-1810–499–301).

### 4.2 Sample collection and preparation

Subjects were requested to refrain from oral hygiene activities at least 2 h prior to sample collection. Specimens were collected from the oral cavity before any dental procedures that could have altered the oral microbiome in a uniform manner by one trained investigator to minimize batch effects, as described previously (Aagaard et al., 2013). Oral specimens were collected in the following order: buccal swabs, saliva, and plaque. Buccal swab samples were collected from the inner buccal mucosa of the right and left cheeks using buccal swab kit. Saliva was collected for 20 min without stimulation. All periodontal probe depths were recorded in advance and subgingival plaque was collected from the two deepest pockets. Venous blood samples were collected under sterile conditions by trained personnel. Stool samples were collected by the subjects themselves using a sterile spatula, placed in a sterile stool sample container, and stored in a freezer until transport to the lab on ice. After collection, all specimens except stool samples were immediately transported to the laboratory and stored at –70°C until DNA extraction. Genomic DNA (gDNA) was isolated from saliva, blood, and stool using a QIAamp DNA mini kit (QIAGEN, Hilden, Germany), from plaque using a QIAamp DNA micro kit (QIAGEN), and from buccal swabs using a QIAamp DNA Blood Mini kit (QIAGEN) according to the manufacturer’s standard protocol. 200 µL of saliva and blood were used. For buccal swab specimens, 400 µL was extracted after gently mixing buccal swab with 1.5 mL PBS in a microcentrifuge tube. For stool specimens, 180–220 mg specimen was mixed with 1 mL InhibitEX buffer (QIAGEN) before processing, and only the supernatant obtained after centrifugation was used. Extracted DNA was prepared for sequencing according to the protocol of the HMP Consortium (Human Microbiome Project C, 2012a; Human Microbiome Project C, 2012b).

### 4.3 16S rRNA sequencing

Each sequenced sample was prepared according to the Illumina 16S Metagenomic Sequencing Library protocol to amplify the V3 and V4 regions (519F-806R). DNA quality was measured using PicoGreen reagent and Nanodrop (BioTek, Winooski, VT, USA). Input gDNA (10 ng) was used for PCR amplification. The barcoded fusion primer sequences used for PCR amplifications were as follows: 519F, 5′-CCTACGGGNGGCWGCAG-3′; 806R, 5′-GACTACHVGGGTATCTAATCC-3′. Then the final purified product was quantified by quantitative PCR using a KAPA Library Quantification kit for Illumina Sequencing platforms (Roche, Mannheim, Germany) according to the manufacturer’s protocol, and the quality was assessed using a LabChip GX HT DNA High Sensitivity Kit (PerkinElmer, Waltham, MA, USA). Next, paired-end (2 × 300 bp) sequencing was performed using the MiSeq platform (Illumina, San Diego, CA, USA).

### 4.4 Data analysis and visualization

The reads were processed using a Divisive Amplicon Denoising Algorithm (DADA) 2-based pipeline conducted within the QIIME2 22.2 platform (Callahan et al., 2016; Bolyen et al., 2019). Briefly, an amplicon sequencing variant (ASV) table was produced by quality-based filtering and trimming, read deduplication, and inference of ASVs, followed by paired-end merging and chimera removal. To correct artifactual biases, the feature tables were normalized by rarefaction. For alpha diversity analysis, indices such as observed features, Shannon’s entropy, Pielou’s evenness, and Faith’s phylogenetic diversity were measured. To estimate the dissimilarities between the microbial compositions of five habitats, beta diversity indices including Jaccard, Bray-Curtis, and unweighted and weighted UniFrac distance matrices were computed. Then, PCoA was applied to visualize the broad trends of the sample dissimilarities, and permutation multivariate analysis of variance (PERMANOVA) of the diversity matrix was conducted to quantify the strengths of the associations between community composition and variables. For taxonomic analysis of microbial composition, the sequences were taxonomically classified against the 99% SILVA rRNA taxonomy using a pretrained scikit-learn naive Bayes machine-learning classifier of the q2-feature-classifier plugin (Quast et al., 2012).

To determine the differentially abundant taxa across body habitats, we conducted ANCOM-BC, which estimates the unknown sampling fractions and corrects the bias induced by their differences through a log-linear regression model (Lin and Peddada, 2020). Moreover, phylogenetic investigation of communities by reconstruction of unobserved states 2 (PICRUSt2), which enables the prediction of microbial functions from 16S marker gene sequences, was performed, and the predicted orthologs were collapsed to KEGG pathways to elucidate any differential microbial metabolism between different habitats (Kanehisa and Goto, 2000; Douglas et al., 2020). The ANOVA-Like Differential Expression tool (ALDEx2) was used for differential abundance testing of the metagenome functions of habitats (Fernandes et al., 2013).

Statistical analysis and data visualization processes other than those described above were performed using *R* software (ver. 4.1.2; *R* Development Core Team, Vienna, Austria). QIIME artifacts were imported into the *R* environment using the *qiime2R* package and converted into phyloseq objects using the *phyloseq* package (McMurdie and Holmes, 2013). To apply conventional statistical techniques to the microbial abundance, CLR transformation was performed. Nonparametric tests were performed using Kruskal–Wallis H-test and two-sided Wilcoxon’s signed-rank test using Benjamini-Hochberg correction. Heatmaps were generated by unsupervised hierarchical clustering using the *pheatmap* function.

## Data availability statement

The datasets presented in this study can be found in online repositories. The names of the repository/repositories and accession number(s) can be found below: https://www.ncbi.nlm.nih.gov/, PRJNA489752.

## Ethics statement

The studies involving humans were approved by the Institutional Review Board (IRB) of Seoul National University Bundang Hospital (IRB number: B-1810–499–301). The studies were conducted in accordance with the local legislation and institutional requirements. The participants provided their written informed consent to participate in this study.

## Author contributions

SO: Conceptualization, Formal Analysis, Investigation, Methodology, Visualization, Writing – original draft, Writing – review & editing. H-JL: Conceptualization, Formal Analysis, Funding acquisition, Investigation, Resources, Supervision, Writing – review & editing. KP: Conceptualization, Formal Analysis, Investigation, Resources, Supervision, Writing – review & editing.
