# Supplementary material for: Metagenomic characterization of the microbiomes in five different body habitats of otherwise healthy individuals with periodontal disease
Source: Front Cell Infect Microbiol. 2023 Sep 13;13:1257816. doi: 10.3389/fcimb.2023.1257816 (PMC10534035; doi:10.3389/fcimb.2023.1257816)
Supplement: Supplementary file 1 [file DataSheet_1.docx]

Supplementary Material

Metagenomic Characterization of the Microbiomes in Five Different Body Habitats of Otherwise Healthy Individuals with Periodontal Disease

Sujin Oh, Hyo-Jung Lee^*^, Kyoung Un Park^*^

*** Correspondence:** H Lee and KU Park: periolee@gmail.com; m91w95pf@snu.ac.kr

# Supplementary Table

**Supplementary Table 1.** The clinical characteristics of otherwise healthy subjects with periodontal disease.

| Subject number | Age | Sex | Ethnicity | Height (cm) | Weight (kg) | BMI (Kg/m2) | Sites with PD  ≥ 6mm | Sites with PD  < 6mm | Mean PD (SD) | Sites with CAL  ≥ 6mm | Sites with CAL  < 6mm | Mean CAL  (SD) | Periodontitis Classification* |  |
| --- | --- | --- | --- | --- | --- | --- | --- | --- | --- | --- | --- | --- | --- | --- |
|  |  |  |  |  |  |  |  |  |  |  |  |  |  |  |
| S-01 | 58 | F | Asian | 155.8 | 56.1 | 23.1 | 1 | 27 | 2.59 (0.93) | 4 | 24 | 2.86 (1.18) | severe |  |
| S-02 | 37 | F | Asian | 162.3 | 52.7 | 20 | 0 | 28 | 2.00 (0.62) | 0 | 28 | 2.18 (0.76) | moderate |  |
| S-03 | 40 | M | Asian | 171.9 | 73.3 | 24.8 | 0 | 28 | 2.15 (0.42) | 0 | 28 | 2.33 (0.63) | moderate |  |
| S-04 | 34 | M | Asian | 172.5 | 54.1 | 18.2 | 0 | 28 | 2.52 (0.81) | 0 | 28 | 2.53 (0.82) | severe |  |
| S-05 | 51 | F | Asian | 151.5 | 44.8 | 19.5 | 0 | 23 | 2.12 (0.40) | 0 | 23 | 2.17 (0.50) | moderate |  |
| S-06 | 52 | M | Asian | 168.8 | 84.3 | 30 | 9 | 19 | 3.04 (1.25) | 10 | 18 | 3.11 (1.31) | moderate |  |
| S-07 | 28 | F | Asian | 172 | 59 | 19.9 | 1 | 27 | 2.49 (0.78) | 1 | 18 | 2.58 (0.74) | moderate |  |
| S-08 | 42 | F | Asian | 156 | 55 | 22.6 | 0 | 24 | 2.46 (0.60) | 0 | 18 | 2.60 (0.65) | moderate |  |
| S-09 | 44 | F | Asian | 159.1 | 49.7 | 19.6 | 0 | 13 | 2.19 (0.56) | 0 | 18 | 2.26 (0.65) | moderate |  |
| S-10 | 42 | F | Asian | 163.8 | 91.5 | 34 | 1 | 27 | 2.62 (1.12) | 4 | 18 | 3.32 (1.22) | severe |  |

Abbreviations: F, female; M, male; BMI, body mass index; PD, probing depth; SD, standard deviation; CAL, clinical attachment loss

*The severity of periodontitis was determined by the Centers for Disease Control and Prevention (CDC)-American Academy of Periodontology (AAP) definition

# Supplementary Figure

**Supplementary Figure 1.** Box plots of the centered log ratio-transformed abundance of 17 selected genera in five habitats of otherwise healthy individuals with periodontal disease.
